# Electronic State Engineering in Perovskite‐Cerium‐Composite Nanocrystals toward Enhanced Triplet Annihilation Upconversion

**DOI:** 10.1002/advs.202305069

**Published:** 2023-10-23

**Authors:** Nan Gong, Runchen Lai, Shiyu Xing, ZhengZheng Liu, Junyao Mo, Tao Man, Zicheng Li, Dawei Di, Juan Du, Dezhi Tan, Xiaofeng Liu, Jianrong Qiu, Beibei Xu

**Affiliations:** ^1^ State Key Laboratory of Extreme Photonics and Instrumentation College of Optical Science and Engineering Zhejiang University Hangzhou Zhejiang 310027 China; ^2^ State Key Laboratory of High Field Laser Physics and CAS Center for Excellence in Ultra‐intense Laser Science Shanghai Institute of Optics and Fine Mechanics (SIOM) Chinese Academy of Sciences (CAS) 201800 Shanghai China; ^3^ Zhejiang Lab 311100 Hangzhou China; ^4^ College of Materials Science and Engineering Zhejiang University 310027 Hangzhou China

**Keywords:** electronic state engineering, triplet energy transfer, triplet‐triplet annihilation, upconversion

## Abstract

Wavelength conversion based on hybrid inorganic–organic sensitized triplet–triplet annihilation upconversion (TTA‐UC) is promising for applications such as photovoltaics, light‐emitting‐diodes, photocatalysis, additive manufacturing, and bioimaging. The efficiency of TTA‐UC depends on the population of triplet excitons involved in triplet energy transfer (TET), the driving force in TET, and the coupling strength between the donor and acceptor. Consequently, achieving highly efficient TTA‐UC necessitates the precise control of the electronic states of inorganic donors. However, conventional covalently bonded nanocrystals (NCs) face significant challenges in this regard. Herein, a novel strategy to exert control over electronic states is proposed, thereby enhancing TET and TTA‐UC by incorporating ionic‐bonded CsPbBr_3_ and lanthanide Ce^3+^ ions into composite NCs. These composite‐NCs exhibit high photoluminescence quantum yield, extended single‐exciton lifetime, quantum confinement_,_ and uplifted energy levels. This engineering strategy of electronic states engendered a comprehensive impact, augmenting the population of triplet excitons participating in the TET process, enhancing coupling strength and the driving force, ultimately leading to an unconventional, dopant concentration‐dependent nonlinear enhancement of UC efficiency. This work not only advances fundamental understanding of hybrid TTA‐UC but also opens a door for the creation of other ionic‐bonded composite NCs with tunable functionalities, promising innovations for next‐generation optoelectronic applications.

## Graphical abstract

It is discovered that electronic state engineering of perovskite nanocrystals leads to size focusing with greatly increased fluorescent quantum yield. This strategy can control triplet population, energy‐level alignment, and exciton lifetime, which significantly enhanced triplet–triplet annihilation upconversion (TTA‐UC) efficiency (≈284%). This work clarifies the functions of electronic state engineering for inorganic sensitizers and TTA‐UC. It will promote the fundamental research of electronic state engineering in energy transfer systems and facilitate the practical applications of TTA‐UC systems.

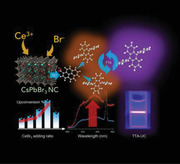

## 1 Introduction

Hybrid triplet–triplet annihilation upconversion (TTA‐UC), which utilizes inorganic materials to sensitize the triplet states of organic molecular emitters through Dexter‐type triplet energy transfer (TET) or sequential, uncorrelated charge transfer process.^[^ ^1^ ^]^ It is widely thought that the two triplet excitons from the nearby emitters can annihilate and form a singlet state with a higher energy photon emission.^[^ ^2^ ^]^ This hybrid system holds great promise for various optoelectronic and biological applications,^[^ ^3^ ^]^ including additive manufacturing,^[^ ^4^ ^]^ solar cells,^[^ ^5^ ^]^ LEDs,^[^ ^6^ ^]^ upconversion lasers,^[^ ^7^ ^]^ photocatalysis,^[^ ^8^ ^]^ and biological imaging.^[^ ^9^ ^]^ In hybrid systems, the inorganic sensitizer, such as semiconductor nanocrystals (NCs) and films, exhibits strong spin‐orbit coupling, a relatively large absorption cross‐section, and tunable absorption spanning from UV to near‐infrared regions. Additionally, they possess a smaller singlet–triplet energy difference compared to organic sensitizers. These characteristics result in significant improvements in anti‐Stokes shifts and UC efficiency while reducing the excitation threshold to subsolar irradiance levels.^[^ ^10^ ^]^ However, these systems face challenges stemming from insufficient energy transfer due to a small population of triplet excitons participating in TET, often caused by nonradiative recombination or competing decay pathways from deep defect states. Additionally, they suffer from a low driving force due to energy level mismatches between the inorganic sensitizer and organic acceptor, as well as a weak coupling strength between the donor and acceptor, which collectively suppress the enhancement of TET and TTA‐UC efficiency.^[^ ^11^ ^]^ Addressing these challenges requires the development of strategies aimed at optimizing the optoelectronic properties of the inorganic sensitizers.

One effective strategy involves engineering the electronic states of inorganic sensitizers to maximize TET efficiency.^[^ ^12^ ^]^ However, this approach has been largely overlooked by the TTA‐UC community in the past, primarily because traditional inorganic sensitizers relied on covalently bonded II–VI or III–V NCs. The inherent self‐purification effect,^[^ ^13^ ^]^ low coordination numbers of cation sites in these NCs,^[^ ^14^ ^]^ and issues related to doping‐induced clustering and phase separation presented formidable challenges.^[^ ^15^ ^]^ Furthermore, precise control over lattice positions and dopant distribution, as well as the management of doping‐induced defect states, proved to be challenging. Consequently, there has been a lack of comprehensive strategies for engineering the electronic states of these NCs to improve TTA‐UC. The significance of electronic state engineering in TTA‐UC was not fully realized until 2018. Researchers discovered that TTA‐UC did not follow the trend of increasing photoluminescence quantum yield (PLQY) in NCs but was closely related to surface defects resulting from the core–shell structure.^[^ ^16^ ^]^ Subsequent studies revealed that surface and extrinsic doped defects could be advantageous for TTA‐UC because these doping‐induced states trapped excitons with improved energy‐level resonance (inhibition of competitive energy transfer) or longer exciton lifetimes.^[^ ^17^ ^]^ Notably, enhanced TTA‐UC was achieved using Au‐doped CdSe,^[^ ^18^ ^]^ indicating that suppressing unwanted pathways and modifying TET‐related optical properties through electronic state engineering could effectively enhance TTA‐UC efficiency.

Among inorganic NC sensitizers, perovskite NCs, particularly CsPbBr_3_, are excellent candidates for investigating TTA‐UC from the perspective of electronic state engineering. These materials are known for their strong defect tolerance,^[^ ^19^ ^]^ high PLQY, bright triplet states,^[^ ^20^ ^]^ and ample room for chemical composition modification.^[^ ^14^ ^]^ Unlike II–VI or III–V NCs, perovskites are ionic crystals whose electronic states can be easily adjusted through external doping^[^ ^12a^ ^]^ or alloying, especially by adding metal halide salts for Pb‐site doping.^[^ ^15^ ^]^ External doping can induce lattice shrinkage to stabilize NCs,^[^ ^21^ ^]^ increase the formation energy of defects, modify the tolerance factor,^[^ ^22^ ^]^ suppress nonradiative channels,^[^ ^23^ ^]^ increase electron density in the conduction band,^[^ ^24^ ^]^ introduce near‐band‐edge defect states,^[^ ^23a^ ^]^ and enable n‐type or p‐type doping,^[^ ^12a^ ^]^ among other effects. In perovskite NCs, these modifications can lead to increased fluorescence lifetimes, enhanced PLQY, and alterations in electronic levels, thereby improving charge transport in related optoelectronic devices.^[^ ^25^ ^]^ Among all the dopants, trivalent lanthanide ions (Ln^3+^) doping has become a research hotspot to modify the bandgap,^[^ ^26^ ^]^ enhance or suppress specific electronic transitions,^[^ ^27^ ^]^ and influence the energy levels and lifetimes of excited states^[^ ^28^ ^]^ for applications in perovskite LEDs^[^ ^29^ ^]^ and solar cells.^[^ ^30^ ^]^ Moreover, previous research has indicated that factors such as the triplet population of the inorganic sensitizer,^[^ ^10a^ ^]^ exciton lifetime,^[^ ^17a^ ^]^ driving force,^[^ ^17b^ ^]^ and coupling strength^[^ ^31^ ^]^ between donor and acceptor significantly impact on TET, ultimately influencing its TTA‐UC efficiency.^[^ ^32^ ^]^ This raises important questions regarding the influence of lanthanide halide‐induced engineering of electronic states in perovskite NCs on the TET process. Specifically, it prompts inquiry into whether this modification affects the population of triplet excitons participating in TET, the energy transfer rate, driving force, coupling strength, and the fundamental mechanisms underlying these influences on the TET process.

In this study, we selected CsPbBr_3_ NCs doped with cerium (Ce) as triplet sensitizers, aiming to investigate the impact of Ce salt incorporation on the electronic states, optoelectronic properties of the NCs, and the TET process. The introduction of cerium bromide (CeBr_3_) to form composite NCs yielded several noteworthy outcomes, including an increase in PLQY, an elevation of the electronic band position, and the manifestation of quantum confinement effects. These changes were conducive to a prolonged single exciton lifetime, an enhanced population of triplet excitons involved in the TET process, and an increased TET rate, which collectively contribute to an amplified TTA‐UC efficiency. By utilizing composite NCs as sensitizers, combined with 1‐pyrenecarboxylic acid (PCA) transmitter ligand and 9,10‐diphenylanthracene (DPA) upconverting organic molecules in a solution, nonlinear enhancement in UC efficiency was achieved with increasing concentration of Ce. Through comprehensive material characterization and time‐resolved spectroscopic analysis, we found that this nonlinear enhancement in TTA‐UC efficiency could be attributed to a modification in the electronic band structure induced by the incorporation of CeBr_3_. This alteration in the electronic band structure also underlies the changes observed in the TET process. By varying the doping concentration, we identified optimized doped samples and compiled the key parameters behind this enhanced performance. Indeed, this research not only marks a significant advancement in the field of enhancing TTA‐UC through the engineering of the electronic states of inorganic NC sensitizers but also introduces a novel class of perovskite‐lanthanide composite NCs. This combination harnesses the unique properties of perovskite materials and lanthanide ions, opening new avenues for the application of NCs in various domains.

## 2 Results and Discussion

**Figure 1:**
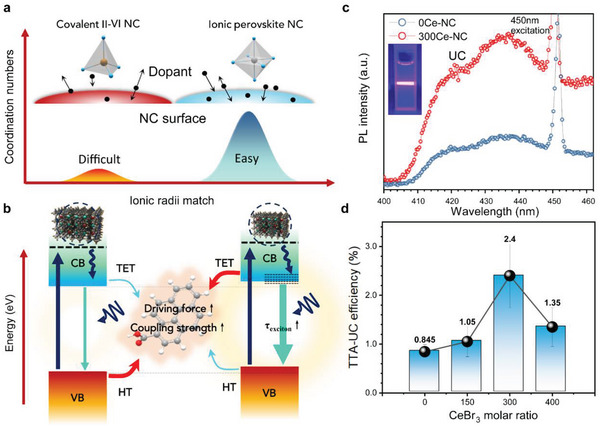
a) The schematic illustrates that higher coordination numbers at cation sites, combined with the minimal mismatch in ionic radii between lanthanide dopants and host cations, result in more efficient doping in halide perovskite (shown in the right inset) compared to covalent II–VI NCs (shown in the left inset). b) Enhancement of hybrid TTA‐UC through engineered electronic states in inorganic sensitizers. This figure highlights the various factors influencing the TET. Notably, the hole transfer (HT) needs to be meticulously controlled to optimize TET. Improvements in PLQY and band‐edge exciton lifetime can further boost TET. Elevating the energy level can increase the driving force behind the TET process, while quantum confinement strengthens the coupling between donor and acceptor molecules. c) Enhanced TTA‐UC was achieved using doped CsPbBr_3_ as sensitizers. Spectra were captured using a 450 nm short‐pass filter. The spectral envelope spanning 400–450 nm corresponds to upconversion emission by DPA, with an excitation at 450 nm. d) TTA‐UC efficiency of NCs‐cerium composite at varying doping concentrations.

**Figure** **1**a provides a comparative overview of the incorporation of lanthanide ions into traditional covalently bonded II–VI and III–V NCs as well as ionic bonded perovskite NCs. It highlights some key challenges associated with covalently bonded NCs (left side of Figure 1a), including low coordination numbers of cation sites, a significant mismatch in ionic radii between the host cations and lanthanide dopants, heterovalent doping‐induced defects, and a limited defect tolerance, all of which resulted in low solubility of lanthanides within these covalently bonded NCs.^[^ ^13^ ^]^ In contrast, perovskite NCs (the right side of Figure 1a) boasted a higher coordination number of cation sites, closely matched ionic radii between the host and lanthanide dopants, and a greater defect tolerance. Consequently, they exhibited a relatively higher solubility for lanthanides compared to covalently bonded NCs. Experimental characterization^[^ ^33^ ^]^ and computational studies^[^ ^34^ ^]^ validated that trivalent lanthanide ions could substitute Pb^2+^ ions in octahedral coordination, forming lattice defects to balance the charge.

As discussed previously and illustrated in Figure 1b, the effective incorporation of lanthanide salts into perovskite NCs emerges as a promising strategy for engineering their electronic states, thereby promoting TET and TTA‐UC achieved through effective perovskite doping, which enhanced PLQY^[^ ^15^ ^]^ and altered the band alignment of the NCs.^[^ ^21a^ ^]^ These electronic state modifications offered several advantages to TET, including the enhancement of band‐edge exciton participation in TET, an increase in the driving force, and the suppression of unwanted competitive pathways. It is noteworthy that heterovalent Ce^3+^ doping could generate additional defect states near the band edge of perovskite NCs,^[^ ^24^, ^35^ ^]^ which acted as bridges facilitating the transfer of triplet excitons from donor to acceptor.^[^ ^17^, ^36^ ^]^ This process resulted in a longer exciton lifetime,^[^ ^21a^ ^]^ size reduction‐induced quantum confinement of NCs through surface passivation, and enhanced chemical potential during synthesis via the addition of extra bromide salts.^[^ ^37^ ^]^ The bright triplet state and strong spin‐orbit coupling of CsPbBr_3_ NCs ensured high radiative recombination, while the high defect tolerance of perovskites enabled the fine‐tuning of exciton lifetimes and facilitated the more favorable transfer of triplet excitons through the TET process. Furthermore, quantum confinement enhanced the coupling strength between donor and acceptor, thereby increasing UC efficiency.^[^ ^31^ ^]^

In this research, we successfully employed this strategy by incorporating CeBr_3_ with the perovskite precursor to create CsPbBr_3_‐Ce composite NCs via the hot‐injection method. Detailed information on the synthesis can be found in the Supporting Information. As shown in Figure 1c, a noteworthy improvement in the TTA‐UC of composite NCs was observed when sensitized with PCA‐DPA, in contrast to undoped perovskite NCs. Of particular significance is the violet upconverted emission, depicted in the inset of Figure 1c. These samples were named based on the molar ratio of CeBr_3_ to PbBr_2_, with designations such as 0Ce‐NC, 150Ce‐NC, 300Ce‐NC, and 400Ce‐NC. Inductively coupled plasma mass spectrometer results indicated insufficient Ce^3+^ dopant incorporation for doping precursor ratios below 100% (Figure S1, Supporting Information). The final atomic ratio of Ce/(Ce+Pb) in CsPbBr_3_ NCs was 29.4% and 38.9% for initial doping precursor ratios of 150% and 300%, respectively. This growth is consistent with expectations, given that we prevented solution supersaturation during high‐temperature synthesis by increasing precursor solubility. Figure 1d summarizes the CeBr_3_ doping molar ratio‐dependent TTA‐UC trends, with efficiency enhancements of up to 2.4% for 300Ce‐NC. It was observed that the slope of upconversion enhancement exhibited a mild increase at low doping levels of 0–150%, but a steep increase at higher doping levels from 150–300%. This suggests the presence of distinct enhancement mechanisms and TET processes within these two doping ranges.

**Figure 2:**
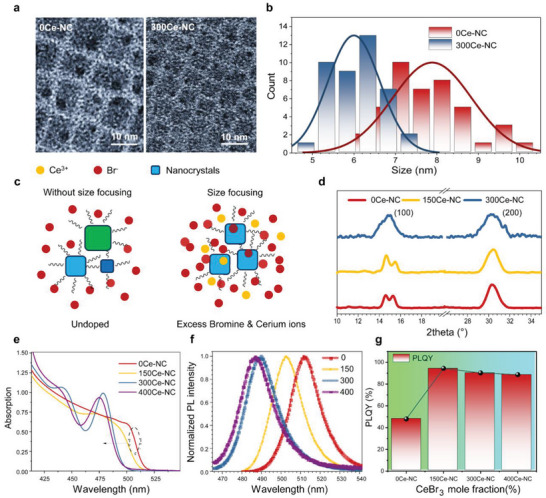
a) TEM images of 0Ce‐NC and 300Ce‐NC. Size‐shrunk can be clearly observed. b) Size‐distribution analysis derived from the TEM images. A slightly tightened size distribution for the 300Ce‐NC suggests a size‐focusing effect. c) Schematic of size‐focusing effect induced by high concentration CeBr_3_ doping. d) XRD patterns show the (100) and (200) planes of doped and undoped NCs. e) Absorption spectra of doped CsPbBr_3_ NCs in a toluene solution, with the undoped NCs included for reference. f) PL spectra of 0Ce‐NC, 150Ce‐NC, 300Ce‐NC, and 400Ce‐NC, respectively. g) PLQY of the stored NCs‐cerium composite with different doping concentrations. Notably, samples doped with CeBr_3_ exhibit a PLQY exceeding 90%.

To quantitatively analyze the impact of electronic state engineering strategies on perovskite NCs, we have systematically compared the structure and optical properties of 0Ce‐NC and CsPbBr_3_‐Ce composite NCs. Transmission electron microscope (TEM) images, as shown in **Figure** **2**a and Figure S2 (Supporting Information), revealed negligible size reduction from 0Ce‐NC to 150Ce‐NC, while further Ce doping resulted in a noticeable size decrease in composite NCs (e.g., 300Ce‐NC). The size distribution also narrowed with increased doping, demonstrating a reduction in the nanoparticle size dispersion (Figure 2b). Importantly, this size reduction and distribution narrowing, which have not been observed in previously reported ZnBr_2_‐doped NCs at the same doping ratio,^[^ ^37^ ^]^ can be attributed to the combined incorporation of Ce^3+^ and excess Br^−^. This modification effectively altered the NCs' surface properties (Figure 2c). The increased feeding of CeBr_3_ led to an excess of bromine, elevating the chemical potential of bromide and causing a shift in the thermodynamic equilibrium.^[^ ^37^ ^]^ Consequently, we observed a reduction in the size of the NCs. To investigate the influence of Ce^3+^ doping on the crystal lattice of NCs, we compare the X‐ray diffraction (XRD) patterns of NCs with varying doping concentrations (Figure 2d). Both doped and undoped NCs exhibited a cubic crystalline structure with diffraction peaks corresponding to the (100) and (200) planes of CsPbBr_3_ cubic crystal. These peaks showed a slight shift to higher angles for 150Ce‐NC and broadening for 300Ce‐NC, consistent with previous reports.^[^ ^24^, ^33^ ^]^

The structural change also modified the optical properties of Ce‐composite NCs. The absorption edge showed a blue shift after doping (Figure 2e). The blue shift for 150Ce‐NC compared to that of 0Ce‐NC was due to the Moss–Burstein effect,^[^ ^38^ ^]^ as there was negligible size reduction for 150Ce‐NC compared to that of undoped NCs. This phenomenon indicated the filling of the conduction band minimum (CBM) by increased electronic donor concentration. Further blue shifts for 300Ce‐NC and 400Ce‐NC were accompanied by well‐defined exciton absorption, primarily attributed to the size reduction. Correspondingly, the fluorescence of the doped composite showed a blueshift from 509 to 488 nm (Figure 2f). In addition to the quantum confinement‐induced shift of PL peaks, Ce^3+^ ions provided additional electronic states near the CBM, which also shifted the PL emission.^[^ ^35^ ^]^ It should be noted that quantum‐confined composites were usually synthesized at low temperatures.^[^ ^31^ ^]^ However, our composites were all synthesized by hot injection at relatively high temperatures with heterovalent Ce^3+^ doping. Therefore, it is highly conceivable that the well‐resolved excitonic absorption produced here was mainly caused by the increased concentration of CeBr_3_. PLQY measurements showed that 150% or higher molar ratios of CeBr_3_‐doped NCs all exhibited >90% and up to 95% PLQY (Figure 2g). Thus, a higher TTA‐UC could be expected for CeBr_3_‐doped composites than that doped by ZnBr_2_, which was usually used in the preparation of perovskite NCs with quantum confinement (Figure S3, Supporting Information). Previous studies have demonstrated that excess bromide ions can also passivate surface bromide vacancies.^[^ ^39^ ^]^ In previous studies, quantum dots synthesized with ZnBr_2_ as an additive displayed PLQYs approaching 60%, requiring additional surface modifications to further improve PLQY to 90%. This indicated that Ce^3+^ ions also play a role in PLQY enhancement. It has also been reported that rare‐earth‐doped perovskite NCs could maintain the spin‐orbit coupling characteristics, and the high PLQY indicated that more band‐edge excitons could participate in the TET process.^[^ ^40^ ^]^ It is highly conceivable that increased PLQY by doping CeBr_3_ enhanced upconversion efficiency because of the increased formation of triplet excitons for 150Ce‐NC. On the other hand, as shown in Figure 1b, the near band edge defect states caused by Ce incorporation could mediate the capture of high‐energy exciton and increase band‐edge excitons,^[^ ^24^ ^]^ which should have promoted the formation of excited triplet in transmitter ligand.^[^ ^17^, ^36^ ^]^ Hence, when increasing CeBr_3_ molar ratio to 150%, the increased PLQY, slight shift of XRD peaks, as well as the negligible decrease of NCs size indicated that more triplet excitons could participate in the TET process by Ce‐composite and the enhanced PLQY, leading to enhanced UC.

It should be noted that when the CeBr_3_ molar ratio was increased above 150%, there was a negligible change of PLQY for 300Ce‐NC and 400Ce‐NC compared to that of 150Ce‐NC, but a large size reduction with the appearance of exciton peaks. Thus, quantum confinement plays a significant role in enhancing the UC process for 300Ce‐NC and 400Ce‐NC.^[^ ^31^ ^]^ The quantum‐confined composite‐NCs offer another dimension to study TET by introducing stronger electronic coupling strength.^[^ ^31^ ^]^ Therefore, the significantly enhanced PLQY and quantum confinement are potential key factors contributing to the improvement of TTA‐UC efficiency. However, it remains to be clarified whether other factors affect the enhancement of TET and which kind of electronic property leads to the nonlinear enhancement of UCQY (Figure 1d).

**Figure 3:**
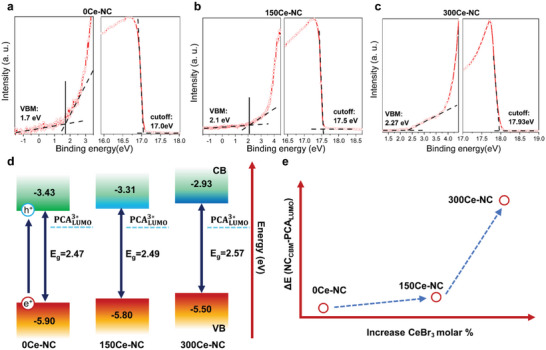
UPS spectra of a) 0Ce‐NC, b) 150Ce‐NC, and c) 300Ce‐NC. d) Illustration of band structure provided by UPS. The up‐lifted CBM and VBM can increase driving force and control competitive pathways, such as HT. e) Increasement of the energy difference between the CBM of NCs and LUMO of PCA by adjusted electronic levels in NCs‐cerium composite.

As shown in **Figure** **3**a–c, ultraviolet photoelectron spectroscopy (UPS) was measured to compare the influence of Ce incorporation on the electronic energy level of NCs. VBM of 0Ce‐NCs was 5.9 eV (Figure 3a). In comparison, the VBM of 150Ce‐NC and 300Ce‐NC shifted upward by 0.1 and 0.4 eV, respectively (Figure 3b,c). The Fermi level also showed an upward shift due to electronic doping.^[^ ^12^, ^41^ ^]^ Considering the Dexter‐type energy transfer process, HT that could compete with this energy transfer was thus partly suppressed in 300Ce‐NC.^[^ ^18^ ^]^ Moreover, the CBM of 300Ce‐NC increased to 2.93 eV, enhancing the driving force of the TET process. Thus, besides the three factors discussed in Figure 2, the formation of composite NCs could also tune the electronic energy level of NCs to modify the driving force or suppress unwanted exciton depletion pathways to enhance TET.

In addition, to verify the upward shift of VBM of the doped NCs and the inhibition of their HT, we utilized 1‐Aminopyrene (AMP), which selectively accepts holes from the NCs due to its lower HOMO level than that of the NCs. Besides, the LUMO level of AMP was higher than that of the NCs (Figure S4, Supporting Information). Therefore, only HT was allowed when the NCs were anchored with 1‐AMP. The PL decay curves of NCs provided additional evidence that the VBM shifted upward because they showed a longer PL lifetime for the doped NCs than the undoped NCs in the NCs‐AMP system. By fitting their PL decay curves with a dual exponential, it was found that the HT efficiency was reduced from 75.4% to 38.8% for 0Ce‐NC and 300Ce‐NC, respectively. Therefore, the position of VBM could be adjusted to a suitable position, and HT can be controlled by CeBr_3_ doping. The above results indicated that changes in the band structure might be one of the significant reasons for the variations in TTA‐UC efficiency. Therefore, precise control of the band structure was an important approach for controlling UC efficiency at the electronic structure level.

**Figure 4:**
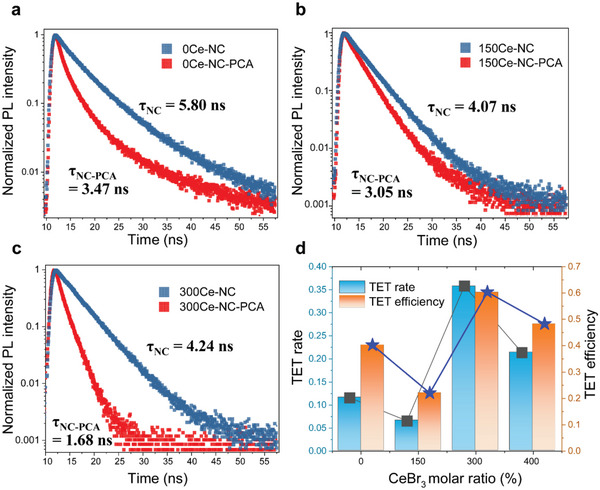
Time‐resolved PL kinetics of a)0Ce‐NC and NC‐PCA complex, b) 150Ce‐NC doped NCs and NCs‐PCA complex, c) 300% doped NCs and NCs‐PCA complex, respectively. d) TET rate and efficiency were extracted from the average lifetime of NCs or NCs‐PCA complex.

As discussed above, three main factors together influenced the TET process, including PLQY, quantum confinement, and energy level change (driving force and HT). Indeed, it is essential to track the PL kinetics of composite NCs and clarify the weight of each factor that influenced TET with increasing doping ratio. To study doping ratio‐dependent TET rate and efficiency change, time‐resolved PL spectroscopy was taken. Typically, the lifetime of NCs‐cerium composite decreased with increasing doping ratio (Table S1, Supporting Information) due to the introduction of near band‐edge‐defect states, which accelerated the radiative recombination.^[^ ^24^ ^]^ All samples exhibited a decrease in fluorescence lifetime after the addition of PCA due to the TET process (**Figure** **4**a–c). It should be noted that zero‐delay PL intensity drop analysis revealed that HT was suppressed at high doping concentrations, consistent with findings in Figure 3d and Figure S5 (Supporting Information).

TET rate and efficiency extracted from the fluorescence lifetime are compared in Figure 4d. While $\Phi_{et}^{{}^{\prime }}$ is defined as TET efficiency, and can be described by^[^ ^17b^ ^,18]^:

(1) $$\Phi_{et}^{{}^{\prime }}=\;1-\tau_{NCs-PCA}/\tau_{NCs},$$

where τ_*NCs*_ is the fluorescence lifetime of the original NCs, and τ_*NCs* − *PCA*_ is the lifetime after the ligand exchange by PCA. Using the lifetime measured above, the TET rate can be calculated by:

(2) $$k_{et}^{{}^{\prime }}=1/\tau_{NCs-PCA}\;-1/\tau_{NCs}.$$

The TET rate and efficiency reach the maximum value for 300Ce‐NC confirming the discussion in Figures 2c and 3 that the quantum confinement due to the decreased size of NCs and the enhanced driving force with electronic level change by Ce^3+^‐doping are two of the factors contributing to enhanced TTA‐UC. It should be noted that both TET rate and efficiency are lower for 150Ce‐NC compared to that of 0Ce‐NC, while TTA‐UC efficiency of 150Ce‐NC is higher than that of 0Ce‐NC. It indicates that the enhanced TTA‐UC for 150Ce‐NC should not be related to quantum confinement‐induced enhanced coupling strength, but to the increased triplet exciton population for 150Ce‐NC as discussed in Figure 2. Whereas for 400Ce‐NC, a drop in both TET rate and efficiency can be observed, and a huge absorption peak appears at ≈400 nm (Figure S6, Supporting Information). This new absorption feature should be related to surface adsorption and causes strong self‐reabsorption, which is detrimental to the TTA‐UC. Therefore, we believe that doping over 400% for further size‐decreased NCs should be inappropriate as TTA‐UC efficiency starts to reduce, and it introduces another channel to deplete UC internal efficiency. By combining Figures 3 and 4 with the trends observed in the zero‐drop decay (Figure S5, Supporting Information), we have identified the nonlinear increase in TTA‐UC efficiency due to the reduction in TET efficiency caused by doping from 0% to 150%. Here, it should be noted that the zero‐delay drop reveals that the proportion of NCs participating in the TET process varies with doping concentration. This result indicates that there might be more exciton consumption in the sub–ps range, leading to the change in η. Therefore, the TET efficiency measured in ns might be underestimated. Hence, there is a need for the TET kinetics regulation in the sub–ps range.

**Figure 5:**
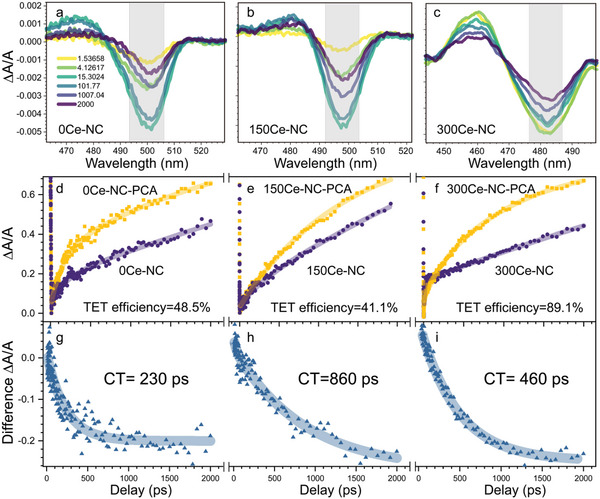
TA spectra of a) 0Ce‐NC, b) 150Ce ‐NC, and c) 300Ce‐NC in a delay time window of 1–2000 ps, which is sufficient to reveal the TET kinetics. The kinetics were obtained from the GSB peak from d) 0Ce‐NC, e) 150Ce‐NC, f) 300Ce‐NC, respectively. The grey area of the spectrum in (a)–(c) indicates where the transient kinetic traces are extracted. Differences in TA kinetics between NCs and NCs‐PCA complex of g) 0Ce‐NC, h) 150Ce‐NC, i) 300Ce‐NC composite illustrate the slow down and re‐acceleration of the charge transfer lifetime. This re‐accelerated lifetime allows more band‐edge exciton in 300% doped composite to participate TET process, resulting in an increased TET efficiency.

To clarify the mechanisms during the early stage of TET, we carried out transient absorption (TA)spectroscopy to monitor the ground‐state bleach (GSB) kinetics in the subnanosecond timescale (**Figure** **5**a–c). Figure 5d–f illustrate the variation in GSB lifetime in NCs when comparing spectra with and without the presence of PCA in transient absorption measurements. The calculated TET efficiency from the lifetime difference confirmed a significant enhancement for 300Ce‐NC and a slight decrease for 150Ce‐NC compared to that of 0Ce‐NC, consistent with the tendency observed in from fluorescence decay curves in Figure 4. We obtained TET kinetics for different doping concentrations by using the GSB difference spectra. It was found that the charge transfer time increased from 230 to 860 ps for 0Ce‐NC and 150Ce‐NC (Figure 5g,h). For 300Ce‐NC, the charge transfer time was shortened to 460 ps (Figure 5i). The slow charge transfer rate in Figure 5h further confirmed the reduced TET efficiency in 150Ce‐NC as shown in Figure 4d. Moreover, the prolonged charge transfer time caused adverse competition with band‐edge radiative recombination, which could reduce the TET efficiency. It further confirmed the findings in Figures 2 and 4, substantiating that the enhancement of PLQY and the near‐band‐edge defect states contributed to enhanced TTA‐UC at low concentrations of doping for 150Ce‐NC.

Additionally, we observed that in NCs with doping exceeding 150% and exhibiting quantum confinement, there was a significant early‐time decay under the same excitation intensity attributed to the multiexciton process induced by the Auger effect. This process was much faster than TET, and the portion of multiexciton to single exciton recombination could also influence the final UCQY. Fortunately, this proportion decreased from 26% to 6% from 150Ce‐NC to 300Ce‐NC (Figure S7, Supporting Information). This result inspired us to fit the GSB and calculate TET efficiency by removing the influence of the early‐stage multiexciton processes. As a result, we obtained TET efficiencies of 48.5%, 41.1%, and 89.1% for 0Ce‐NC, 150Ce‐NC, and 300Ce‐NC, respectively. This tendency matched the result in Figure 4d. The estimation of single‐exciton lifetime also implied that, with increasing doping concentration, the single‐exciton lifetime of NCs increased from 2918 ps for 0Ce‐NC to 8273 ps for 300Ce‐NC (Table S2, Supporting Information). A longer single exciton lifetime was beneficial for increasing TET efficiency since it reduced the competition from radiative recombination to that of the TET process. At this stage, the changes in PLQY, TET efficiency, and charge transfer time did not accurately reflect the true trend in TTA‐UC efficiency changes. However, back to zero‐delay drop analysis, we discovered that after considering the contribution of η to TET, the improvement ratio of TTA‐UC was closely aligned with $\eta \Phi_{et}^{{}^{\prime }}$. Therefore, the contribution of η to TET must be taken into account.

In a hybrid TTA‐UC regime, the efficiency of UC emission can be expressed by:^[^ ^11^ ^]^

(3) $$\mathrm{UCQY}\%\;=\frac{1}{2}\;\eta f\Phi_{em}\Phi_{et}^{{}^{\prime }}\Phi_{et}^{{}^{\prime \prime }}\Phi_{TTA},$$

where the sensitizer mainly influences η (the fraction of NCs that participate in TET) and $\Phi_{et}^{{}^{\prime }}$ (TET from sensitizer to transmitter). *f*, Φ_*em*_, $\Phi_{et}^{{}^{\prime \prime }}$, Φ_*TTA*_, are statistical weight, emitter PLQY, TET from transmitter to emitter, and TTA efficiency, respectively, and should be the same in our investigated systems. For 150Ce‐NC, we can learn from TA results that the $\Phi_{et}^{{}^{\prime }}$ efficiency is reduced from 48.5% to 41.1% based on single exciton lifetime estimation, which we found the result obtained more closely to the TTA‐UC enhancement ratio. As shown in Figure S8 (Supporting Information), the zero‐drop measurement demonstrates that η is also enhanced in the case of 150Ce‐NC. Therefore, the global TET efficiency should be $\Phi_{et}^{g}=\;\eta \Phi_{et}^{{}^{\prime }}$.^[^ ^18^ ^]^ Amazingly, the enhancement of TTA‐UC is almost equal to the improvement of the actual TET efficiency, where the $\Phi_{et}^{g}$ for 0Ce‐NC and 150Ce‐NC are 14.7% and 18.9%, respectively. This improvement has a factor of ≈1.3, which is close to the TTA‐UC enhancement with a factor of ≈1.2. This relationship still holds true in 300Ce‐NC, and the deviation in this enhancement ratio might arise from the loss of PLQY due to ligand exchange. Overall, our results substantiate the positive effect of electronic state engineering strategy on TTA‐UC through both theoretical analysis and experimental validation, and the fraction η of participating TET is a crucial factor and should be carefully controlled in composite NCs. As a result, NCs of high PLQY and the increased single exciton lifetime have adequate band‐edge triplet excitons to participate in TET, while near band‐edge defect states, appropriate energy level matching, and quantum confinement offered better driving force and electronic coupling for TET, all together ensure efficient TTA‐UC.

Finally, it is worth emphasizing that for electronic state engineering, it is not only necessary to improve the PLQY and single‐exciton lifetime of the sensitizer but also to properly control the energy level matching, energy transfer rate, and coupling strength. The interplay of these parameters will comprehensively determine whether electronic state engineering can effectively improve the efficiency of TTA‐UC. This work revealed the importance of electronic state engineering strategies for hybrid TTA‐UC. In addition, the fraction of NCs participating in TET still needs to be further determined and should be related to the PLQY of NCs, energy‐level matching, and fluorescence lifetime. Meanwhile, the different solubilities of metal halide salts in the precursor solution significantly affect the optical properties of NCs and their triplet sensitization.^[^ ^12b^ ^]^ Electronic state engineering for boosting TTA‐UC still requires a systematic examination with various doping strategies. Detailed characterization of the structure and properties of hybrid TTA‐UC systems and ultrafast kinetics are needed to reveal their effects on triplet sensitization.

## 3 Conclusion

In conclusion, electronic state engineering through perovskite‐Ce composite‐NCs significantly promoted the efficiency of TTA‐UC. Utilizing Ce‐composite CsPbBr_3_ as sensitizers, the UC efficiency was increased from 0.85% to 2.40%. This improvement in efficiency was attributed to the enhancement in PLQY, the elevation of electronic levels, and the coupling strength provided by quantum confinement. Besides, the nonlinear shifts of electronic levels for both the CBM and VBM influenced the charge transfer time. Moreover, the optimized photoluminescence of the NCs also extended the singlet exciton lifetime. Overall, the different weights of the functions of these factors at different doping concentrations contributed to the nonlinear enhancement in TET efficiency. However, several issues still need to be addressed: i) The precise characterization of heterovalent doping remains challenging. This work can only demonstrate that doping has produced some positive effects, while the influence of heterovalent doping on excited‐state dynamics can still only be explained with the assistance of existing theoretical results. ii) Further evidence is warranted to support the positive effect of near‐band‐edge defect states on TTA‐UC, such as separately measuring the TET efficiency of band‐edge states and defect states. Besides, the specific defect states and their distribution remain to be explored, requiring the combination of high‐level DFT calculations, electronic structure characterization, and transient dynamics to systematically elucidate the regulatory role of these defect states on TET. iii) According to the above analysis, it is highly conceivable that the charge transfer time, single‐exciton lifetime, and η play an important role at low and high doping concentrations, respectively. However, the contribution of each factor to the improvement of TTA‐UC should be accurately estimated, and a comprehensive investigation of other heterovalent ions is also required. This work opens a door for manipulating the electronic properties of NCs through heterovalent doping, leading to the creation of composite NCs that enable enhanced control and improvement of processes like TET and TTA‐UC. This strategy can in principle be adapted to a variety of material and device designs whether upconversion or downconversion, including solar cells, LEDs, upconversion lasers, photocatalysis, additive manufacturing, and so on.

## 5 Conflict of Interest

The authors declare no conflict of interest.

6

## Supporting information

Supporting Information

## Data Availability

The data that support the findings of this study are available from the corresponding author upon reasonable request.
