# Supplementary material for: Electronic State Engineering in Perovskite‐Cerium‐Composite Nanocrystals toward Enhanced Triplet Annihilation Upconversion
Source: Adv Sci (Weinh). 2023 Oct 23;10(34):2305069. doi: 10.1002/advs.202305069 (PMC10700194; doi:10.1002/advs.202305069)
Supplement: Supplementary file 1 — Supporting Information [file ADVS-10-2305069-s001.pdf]

ADVANCED SCIENCE
Open Access

Supporting Information

for *Adv. Sci.*, DOI 10.1002/advs.202305069

Electronic State Engineering in Perovskite-Cerium-Composite Nanocrystals toward Enhanced Triplet Annihilation Upconversion

*Nan Gong, Runchen Lai, Shiyu Xing, ZhengZheng Liu, Junyao Mo, Tao Man, Zicheng Li, Dawei Di, Juan Du, Dezhi Tan, Xiaofeng Liu, Jianrong Qiu and Beibei Xu**

Experimental

Materials

Oleic acid (technical grade 90%), OAm, (technical grade 70%), and octadecene (ODE, technical grade 90%) were purchased from Sigma Aldrich, Cesium carbonate (Cs_2CO_3, 99.99%, metals basis), lead bromide ($PbBr_2$, 98%) were purchased from Aladdin. Cerium bromide ($CeBr_3$, ultra dry, 99.9%) was purchased from Alfa Alesa. Toluene (99%, anhydrous) was purchased from Yonghua chemical. Methyl acetate (99%, SuperDry, water ≤ 30 ppm, J&KSeal) was purchased from J&K Scientific. 1-Pyrenecarboxylic Acid (1-PCA, 97%), 1-Aminopyrene (AMP, 98%), and 9,10-Diphenylanthracene (DPA, 98%) were purchased from Aladdin. All chemicals were used as procured without further purification.

Synthesis of NCs-cerium composite with different sizes

Preparation of cesium oleate solution: Cs_2CO_3 (0.814 g) was loaded into 250 mL 3-neck flask along with ODE (30 mL) and oleic acid (2.5 mL), dried for 1 h at 120 °C, and then heated under nitrogen atmosphere to 150 °C until all Cs_2CO_3 reacted. The solution was kept at 100 °C before injection.

Preparation of precursor solution: First put the mixture of $PbBr_2$ (0.188mmol) and ODE (5ml) into a 25mL three-necked flask and degas for 60 minutes. At the same time, the OAm needed for the experiment was also degassed until it was added to the precursor solution. Different molar ratios of $CeBr_3$ (0-400% to that of $PbBr_2$) were dissolved in the OA solution in advance and stirred at 70°C until completely dissolved. The precursor solution was prepared by injecting pre-dissolved $CeBr_3$: OA solution (1 ml) and degassed OAm (1 ml) into a 25 ml three-neck flask containing $PbBr_2$ mixed with ODE and reacted at 120°C for 10 minutes under nitrogen atmosphere. It should be noted that due to the solubility of $CeBr_3$, precipitation will occur with the increase of the molar ratio, which will make it difficult to control the doping concentration of the final synthesized NCs. To ensure sufficient dissolution, OA and OAm were increased to 2ml when feeding $CeBr_3$ higher than 150% molar ratios.

Hot injection reaction: The reaction was finally carried out by hot injection. After all the precursors were completely dissolved, the precursor solution was rapidly heated to 190°C under nitrogen atmosphere with vigorous stirring. Take 0.4 ml of Cs-oleate solution and quickly inject it into the vigorously stirred precursor solution, and it can be observed that green luminescent perovskite NCs are rapidly generated. After maintaining the temperature for 1 min, put the three-necked flask into a cold-water bath. The reaction was quenched, and the reaction was completed when the temperature dropped below 30°C.

Purification: the crude solution was centrifuged at 7000 rpm for 5 min to remove the unreacted salts. Both precipitate and supernatant were retained and were subjected to subsequent purification respectively. This is because as the molar ratio of doping increases, the size distribution of nanocrystals will shift to small sizes, resulting in a decrease in the concentration of large-sized nanocrystals that can be generated, and the extraction of smaller-sized NCs need additional steps. The collected precipitate was dispersed with 5 ml of toluene, and then centrifuged at 12,000 rpm for 5 minutes, and the obtained supernatant was the larger size NCs synthesized at this temperature. It should be noted that when feeding at 0-100% molar ratio, the content of localized quantum dot is negligible. Then, the supernatant of the crude solution was added to ~8ml of methyl acetate until the solution was slightly crude, then centrifuged at 10,000 rpm for 5 minutes to get the precipitate and redispersed into ~5ml of toluene solution. The resulting solution was perovskite NCs with stronger quantum confinement.

Ligand exchange on NCs: The process of replacing the native OA ligand with PCA transmitters was carried out in a glovebox. For samples with varying dopant concentrations, 100 µL of the original solution was diluted with dry toluene to achieve an absorbance of approximately 0.2 in the cuvette with 10 mm optical path. Subsequently, 2 mL of this solution was mixed with 2 mg of PCA and stirred at room temperature overnight. Insoluble ligands were removed using a 0.22-micron syringe

filter. Then, 80 μL of DPA toluene solution (10 mg/mL) was added to obtain the final upconversion samples, which were transferred to hermetically sealed cuvettes for analysis.

Characterizations

TEM images were acquired on a Hitachi HT-7700 transmission electron microscope with an accelerating voltage of 120 kV. The X-ray diffraction results were recorded using Rigaku Smartlab X-ray diffractometer equipped with 9kV Cu Kα source. The ultraviolet−visible (UV−vis) absorption spectra were collected by a UV-Vis-NIR spectrometer (UH-5700, Hitachi, Japan). The absolute PLQY of NCs-cerium composite toluene solutions was obtained by a UV-NIR absolute photoluminescence quantum yield spectrometer (Quantaurus-QY Plus C13534-12, Hamamatsu Photonics, Japan), where the error is within 1%, all solutions were properly diluted to ensure little reabsorption effect. The ultraviolet photoelectron spectra (UPS) were carried out with a 21.2 eV He-Iα source and a −9.8 eV bias. Samples for UPS were prepared by drop casting doped and undoped NCs on a 10 mm × 10 mm ITO substrate. The ultrafast transient absorption (TA) uses a femtosecond amplified Ti:sapphire laser source with pulse duration of ∼100 fs and repetition rate of 1 kHz. The laser output is divided into two parts, and the major part is used to generate pump pulses at 450 nm (8μJ/cm^2) in an optical parametric amplifier. The pump beam is modulated at 500 Hz by a mechanical chopper. Another part of the laser is used to generate white light through a CaF_2 crystal plate. The white light can be focused onto the sample by an off-axis parabolic mirror and detected by a silicon detector. The time delay between pump and probe beams is controlled by a motorized stage. The PL decay curves were monitored at the PL center wavelength of each sample using FL920 fluorescence spectrometer (Edinburgh Instruments, UK) under ambient conditions with 470 nm picosecond laser excitation. The data collected was based on time-correlated single photon counting (TCSPC). The excitonic absorptions of all the TTA-UC solution samples were controlled around ~0.2 (10 mm optical path). PL lifetime is obtained by double exponential fitting. All the element ratios were determined using an Agilent 720ES

inductively coupled plasma−optical emission spectrometer (ICP−OES). The TTA-UC upconversion measurement was carried out with a home-made upconversion measurement system with a power-tunable cw laser of 450 nm. The absolute and filtered fluorescence (450 short-pass filter) were collected by an Ocean-Optics USB2000+ spectrometer. All the excitation thresholds were extracted through power-dependent upconversion fluorescence measurements. The photo of upconversion fluorescence was taken by mobile phone through a 425nm short-pass filter.

The efficiency and excitation threshold are analyzed by integrating the DPA fluorescence spectra. Since the upconverted emission of DPA is a linear optical process, the effective range over which the upconverted emission can be integrated is from 400 nm to ~440 nm. By comparing the DPA spectrum excited by down-conversion, the spectral integral intensity in this interval plus the loss of filter transmittance accounts for 52.5% of the overall spectral intensity. As a result, the recovered integrated upconversion intensity was used to calculate the TTA-UC efficiency and excitation threshold. For the estimation of the TTA-UC efficiency, the following equation was used:

$$\Phi'_{UC} = \Phi_{std}\left(\frac{A_{std}}{A_{UC}}\right)\left(\frac{F_{UC}}{F_{std}}\right)\left(\frac{I_{std}}{I_{UC}}\right)\left(\frac{\eta_{UC}}{\eta_{std}}\right)^2 \quad (1)$$

where *F, A, I,* and η represent the quantum yield, absorbance at the excitation wavelength, integrated photoluminescence spectral profile, excitation intensity, and the refractive index of the solvent, respectively. Subscripts std and UC are Coumarin 307 and sample, respectively. Coumarin 307 was used as the reference whose PL quantum yield is 75% in ethanol. To better compare with the upconversion efficiency reported in recent studies, a pre-factor 2 is not introduced, so that the mentioned TTA-UC efficiency in the main text should refer to the unnormalized upconversion efficiency.

Table S1. Fitting parameters of time-resolved PL lifetime from $CsPbBr_3$ NCs with different $CeBr_3$ doping mole ratios.

Samples	A1	t1(ns)	A2	t2(ns)	Avg.(ns)	TET rate (ns^{-1})	TET eff.
0%	0.86	3.12	0.26	8.91	5.80	0.12	0.40
0%-PCA	1.62	1.07	0.22	6.45	3.47		
50%	0.84	4.26	0.06	10.82	5.27	0.08	0.30
50%-PCA	0.78	1.82	0.35	5.17	3.70		
100%	0.85	4.61	0.06	12.71	5.92	0.03	0.15
100%-PCA	1.17	1.23	0.36	7.17	5.04		
150%	Single exponential				4.07	0.08	0.25
150%-PCA	0.97	1.79	0.18	5.30	3.05		
200%	Single exponential				4.52	0.16	0.42
200%-PCA	1.42	1.18	0.35	4.21	2.60		
300%	Single exponential				4.24	0.36	0.60
300% -PCA	0.022	5.861	2.032	1.509	1.68		
400%	Single exponential				3.90	0.19	0.42
400% -PCA	0.237	0.751	0.811	2.394	2.26		

Table S2. Single-exciton emission lifetime fitting. The fitting lifetime increases with higher doping ratio. These fittings were conducted by removing the early 100ps kinetics in transient absorption, which was believed that the multiexciton Auger process had completely decayed. The resulting kinetics can be fit by single exponential, representing the single-exciton lifetime.

Doping ratio	0%	150%	200%	300%
$\tau_{single\text{-}exciton}$(ps)	2918	3328	5993	8273

Supplementary figures

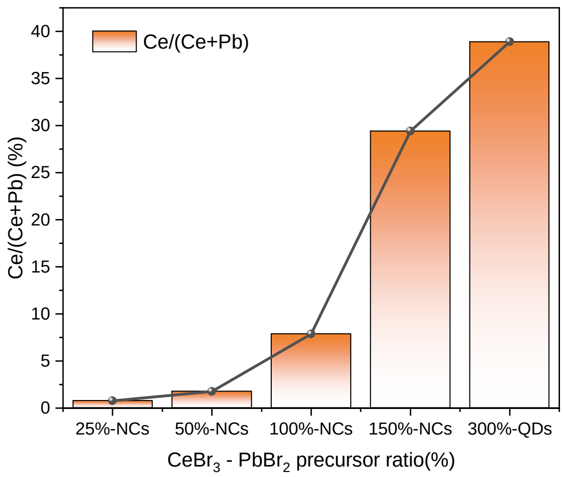

Figure S1. ICP-MS results of 25%, 50%, 100%, 150%, and 300% NCs-cerium composite, respectively.

As discussed above and in the main text, for the $CeBr_3$ feeding ratio lower than 100%, there is no quantum confinement and the size change of NCs is not significant. ICP-MS results showed that the Ce/(Pb+Ce) molar ratio is much lower than that using more than 150% $CeBr_3$ feeding. Thus, the decrease of TTA-UC should be attributed to the effects of passivation caused by excess Br^-, indicating that the low concentration of the doped Ce^{3+} ions and high concentration of Br^- ions may be one of the reasons that lead to the low efficiency of TTA-UC at the doping concentration of 50%. Although surface passivation brought by Br^- and Ce^{3+} ions are beneficial for radiative recombination, we clarify that the Br^- passivation may negatively influence on TTA-UC. In contrast, Ce^{3+} is much more difficult to incorporate into the $CsPbBr_3$ lattice than Br^-, which is why Ce concentration rises significantly and positively influences the TTA-UC after doping with 150% or more $CeBr_3$.

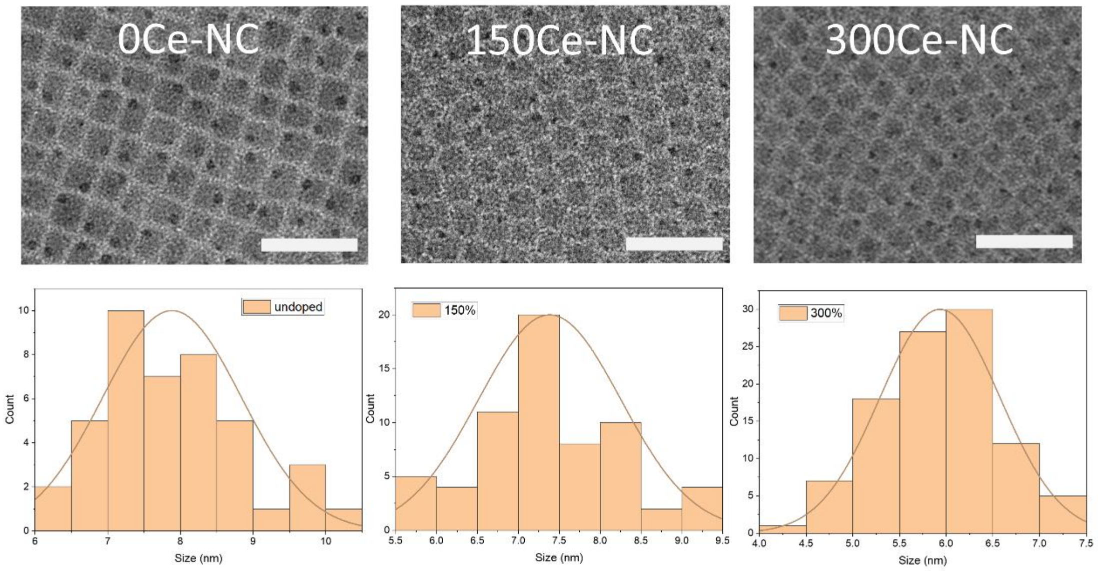

Figure S2. TEM images of NCs-cerium composite with doping ratio at 0%, 150%, 300%, respectively.

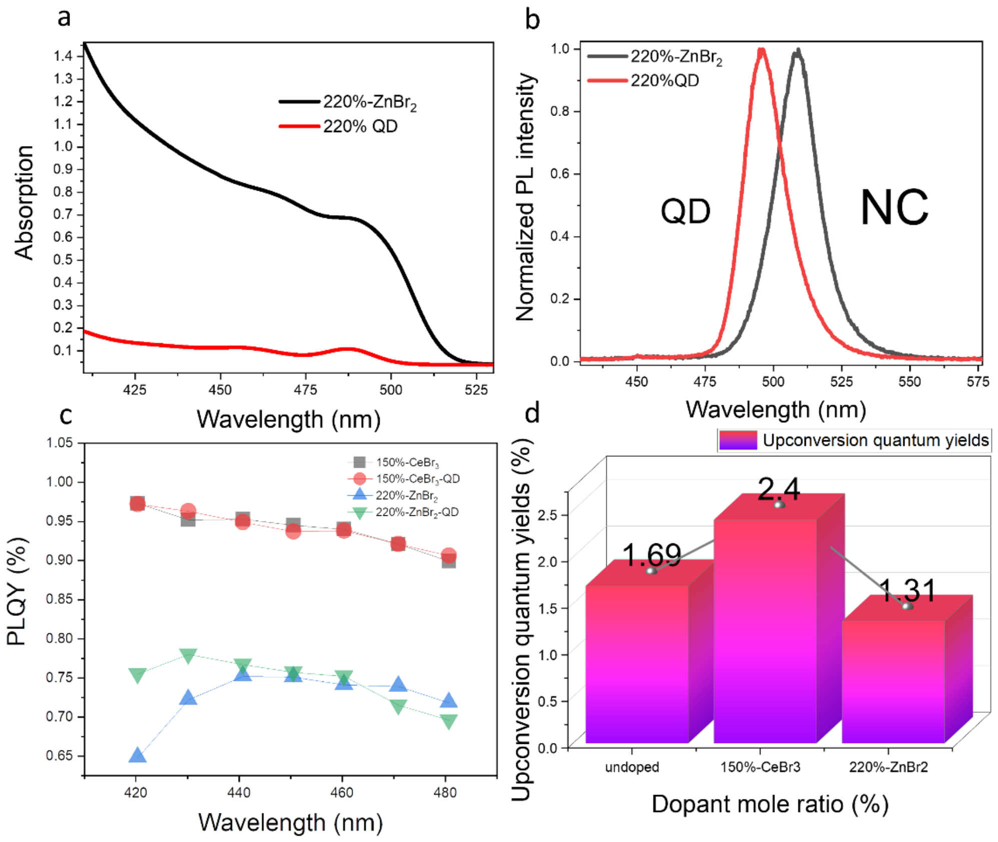

Figure S3. 220% Zinc bromide ($ZnBr_2$, anhydrous, 98%, Alfa Alesa) doped $CsPbBr_3$ NCs and QDs with the same bromide feeding as that of 150Ce-NC. As discussed in the main text, size focus effect is weak for this bromide concentration. Therefore, small amounts of quantum confined NCs can be generated. All NCs were dispersed with same amount of toluene in (a). Although blue shift can be found in PL spectra of $ZnBr_2$ doped samples (b), the PLQY of their stored solution exhibit ~20% PLQY lower than that of the 150Ce-NC. As expected, decreased TTA-UC efficiency was observed for the 220% $ZnBr_2$ doped $CsPbBr_3$ NCs. These results confirm our speculation that the excessive bromine ions can passivate the surface of NCs but will have negative effects on TTA-UC. In contrast, Cerium ions further increases band-edge states, which make PLQY over 90%.

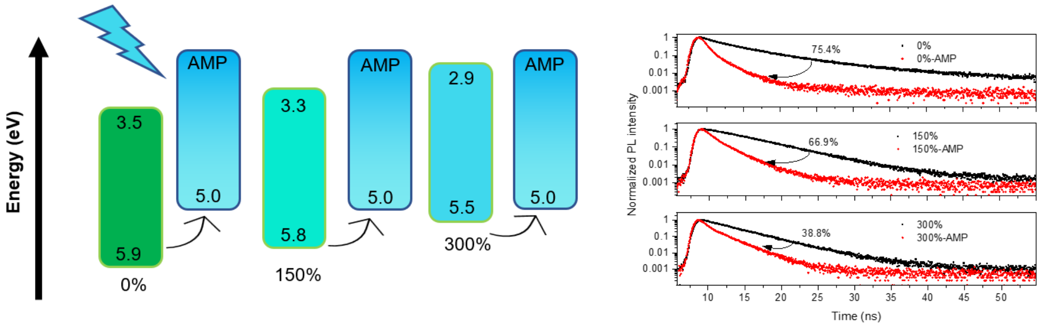

Figure S4. Illustration of band structure determined by UPS. HOMO level of AMP was also shown because AMP was selected as hole-transfer acceptor.

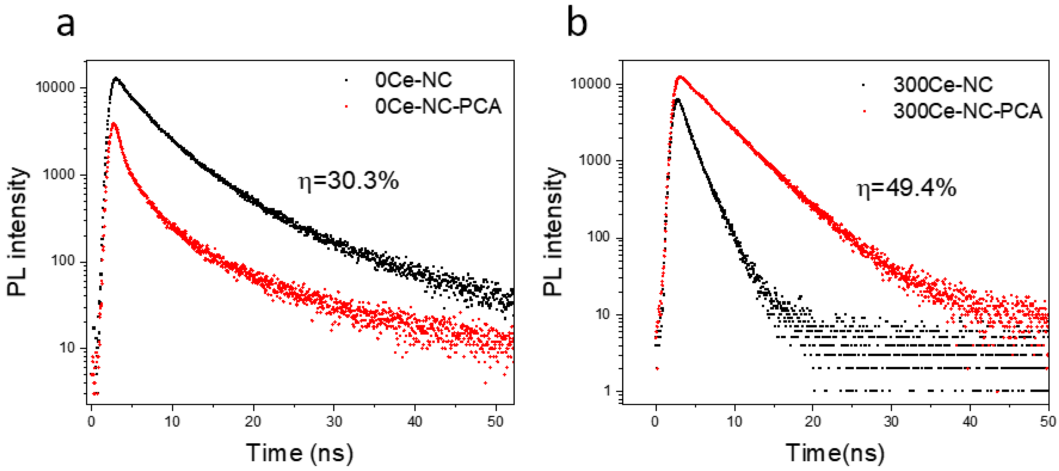

Figure S5. Zero-delay PL intensity drop analysis of (a) 0Ce-NC, and (b) 300Ce-NC.

By zero-delay PL intensity drop analysis, it is found that hole transfer was suppressed at high doping concentration, and the η increases. η can represent the proportion of exciton participated TET[1]. When considering the product of η and TET efficiency, the actual TET efficiencies Φ'_{et} obtained for undoped and doped NCs are 44.5% and 14.7% respectively. The ratio (3.00) is very close to the ratio of TTA-UC efficiencies (2.84) using undoped and doped composite. However, an excessively rapid hole transfer process may have no benefit for the slower direct Dexter transfer process. Previous research has also indicated that hole transfer mediated TET can lead to significant energy loss, which is detrimental to TTA-UC[2]. Furthermore, we have obtained results without a zero-delay drop in 200% doped NCs-cerium composite, but its TTA-UC performance is not satisfactory. This suggests that if hole transfer is completely suppressed, radiative recombination will compete with TET, leading to a decrease in TET efficiency. It also indicates that the rates of hole transfer and radiative recombination need to be precisely controlled through accurate electronic state engineering. They should neither be too fast nor too slow in order to achieve the optimal effect of hole transfer-mediated TET.

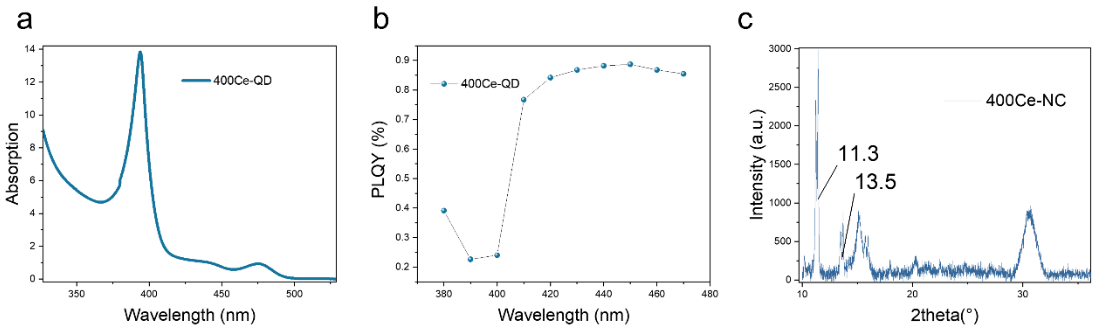

Figure S6. (a) sharp absorption peak can be observed in 400Ce-NC below 400 nm. We have experimentally checked this absorption peak and it originated from the excess $CeBr_3$. After washing, the optical density of this peak can be reduced. (b) This electronic transition is harmful to both PLQY and TTA-UC due to the strong re-absorption. Therefore, we believe that further doping NCs with over 400% $CeBr_3$-$PbBr_2$ should not be a wise choice for enhancing TTA-UC. (c) XRD pattern of 400Ce-NC, where an obvious peak at 11.3° and 13.5° can be detected. This may relate to Cs3CeBr6 reported previously[3].

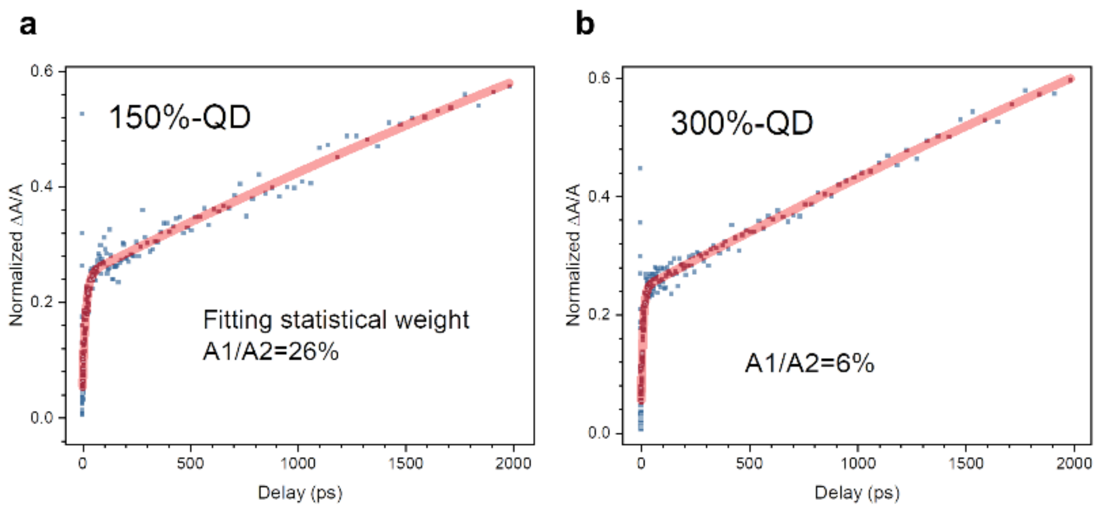

Figure S7. Auger effect in the early time in TA, and the relative transient kinetics fitting for (a) 150Ce-NC, and (b) 300Ce-NC.

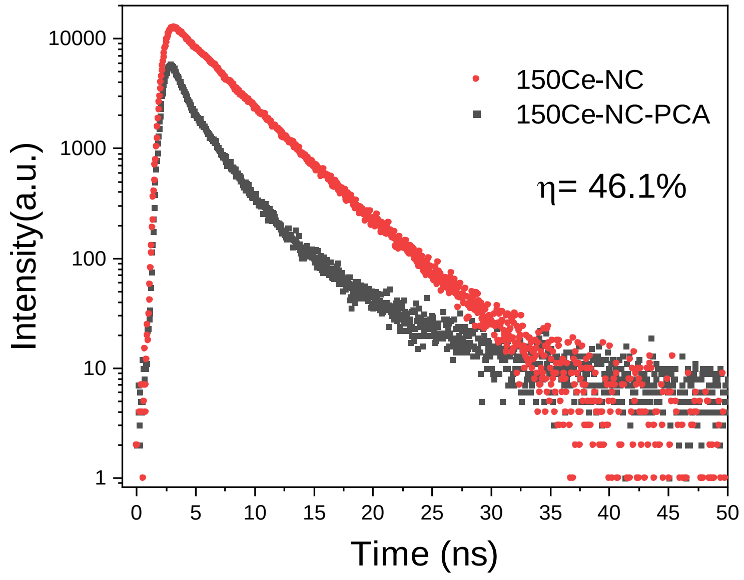

Figure S8. Zero-delay drop of 150Ce-NC. Combine with Figure S5, we can find that the η increases with a factor of ~1.78. The overall(actual) TET efficiency for 0Ce-NC and 150Ce-NC are 14.6% and 18.9%, extracting experimental data from Figure 5 and Figure S5.

References:

[1] A. Ronchi, C. Capitani, V. Pinchetti, G. Gariano, M. L. Zaffalon, F. Meinardi, S. Brovelli, A. Monguzzi, *Adv Mater* **2020**, 32.

[2] X. Luo, Y. Y. Han, Z. W. Chen, Y. L. Li, G. J. Liang, X. Liu, T. Ding, C. M. Nie, M. Wang, F. N. Castellano, K. F. Wu, *Nat Commun* **2020**, 11.

[3] L. Wang, Q. X. Guo, J. S. Duan, W. W. Xie, G. Q. Ji, S. R. Li, C. Chen, J. H. Li, L. B. Yang, Z. F. Tan, L. Xu, Z. W. Xiao, J. J. Luo, J. Tang, *Acs Energy Lett* **2021**, 6, 4245.
